# Rare Periodontal Ligament Drainage for Periapical Inflammation of an Adjacent Tooth: A Case Report and Review of the Literature

**DOI:** 10.1155/2014/879562

**Published:** 2014-12-21

**Authors:** Hongmei Guo, Wei Lu, Qianqian Han, Shubo Li, Pishan Yang

**Affiliations:** ^1^Department of Periodontology, School of Stomatology, Shandong University, 44-1 Wenhuaxi Road, Shandong Province, Jinan 250012, China; ^2^Key Laboratory of Oral Biomedicine of Shandong Province, School of Stomatology, Shandong University, Jinan 250012, China

## Abstract

*Aim*. To report a case with an unusual drainage route of periapical inflammation exiting through the gingival sulcus of an adjacent vital tooth and review probable factors determining the diversity of the discharge routes of periapical inflammation. *Summary*. An 18-year-old male patient presented with periodontal abscess of tooth 46, which was found to be caused by a periapical cyst with an acute abscess of tooth 45. During endodontic surgery, a rarely reported drainage route for periapical inflammation via the gingival sulcus of an adjacent vital tooth was observed for the first time. Complete periodontal healing of the deep pocket of tooth 46 and hiding of the periapical cyst of tooth 45 followed after root canal treatment and periapical surgery with Bio-Oss Collagen implantation on tooth 45. The drainage routes of periapical inflammation are multivariate and the diversity of drainage pathways of periapical inflammation is mainly related to factors such as gravity, barriers against inflammation, and the causative tooth itself.

## 1. Introduction

A sinus tract of endodontic origin is a drainage duct resulting from the suppuration produced by the periapical inflammatory process, which frequently occurs secondary to pulpal degradation followed by invasion of numerous oral microorganisms, which leads to an inflammatory lesion in the periapical periodontium of the affected tooth [1, 2]. The microbiologically induced inflammation may either remain localized within the alveolar bone or penetrate the alveolar bone to spread through the least resistant tissue plane, creating a convoluted drainage duct [2, 3].

Such drainage ducts originating from periapical inflammation can be generally divided into two categories, extraoral or intraoral sinus tracts. An extraoral stoma often presents with a cutaneous sinus tract appearing on a patient's chin, cheek, neck, and perinasal areas [1, 3–5]. It can occasionally penetrate the floor of the maxillary sinus and nasal cavity to manifest as maxillary sinusitis or a nasal fistula [6, 7]. In addition, it also can involve the fascia to cause infections of fascial spaces [8]. Compared with the extraoral drainage sinus, the intraoral one is much more common. The drainage sinus was commonly seen on the facial or palatine mucosa and the gingival sulcus opposite the causative tooth, but at times, it may be some distance from the source and close to other uninvolved teeth [9, 10]. Occasionally, it can be found at the gingival sulcus of a tooth adjacent to the causative tooth [11].

Due to the diversity of drainage pathways of periapical inflammation, there have been numerous misdiagnosed cases, especially for a sinus tract distant from the causative tooth. It has been estimated that half of the patients with extraoral sinus tracts are submitted to multiple surgical operations and long-term application of antibiotics before a correct diagnosis is established [12]. Moreover, a misdiagnosis for causative tooth can frequently lead to the unnecessary endodontic therapy of an uninvolved tooth, especially when the sinus tract is close to other healthy teeth [13, 14]. It is critical to make a correct diagnosis. Until now, the drainage pathways of periapical inflammation exiting through the facial or palatine mucosa are well documented and those exiting through the extraoral skin are also well reported. However, little information is available about ectopic sinus tracts of pulpal origin exiting through the gingival sulcus of an adjacent tooth, which may be mistaken for periodontal lesions [10, 15].

The case reported demonstrates a rare discharge route for periapical inflammation exiting through the gingival sulcus of an adjacent periodontally healthy tooth.

## 2. Case Report

### 2.1. Diagnosis

A healthy 18-year-old male patient attended a department of periodontics in a large teaching hospital, with a chief complaint of gingival swelling producing moderate discomfort for about one week, which was located in the mandibular right buccal region.

**Figure 1:**
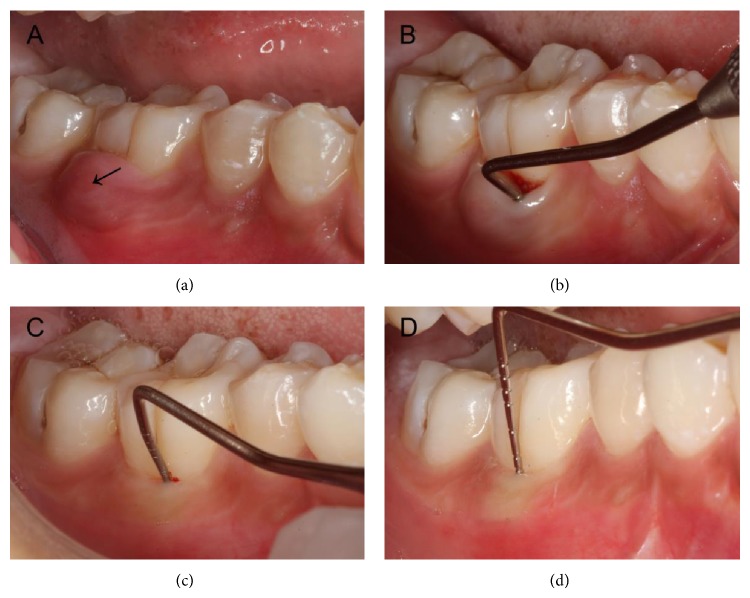
Clinical intraoral photographs. (a) Gingival swelling over the buccal region of tooth 30 (see the dark arrow) and healthy gum of tooth 29. (b) A 10-mm pocket in the buccal furcation of tooth 30 at the initial visit. (c) A 4-mm furcal pocket of tooth 30 in three-month follow-up after operation. (d) At 7-month recall, the furcal probing depth of tooth 30 lessened to about 2 mm.

**Figure 2:**
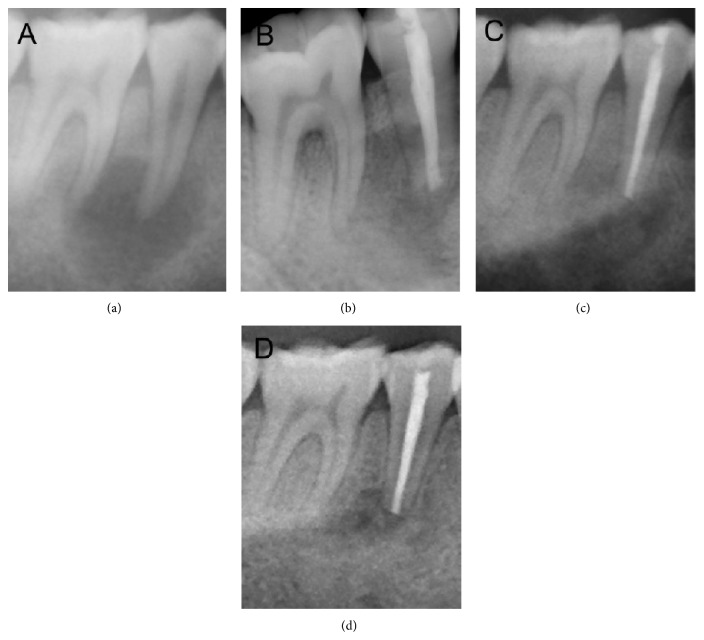
Radiographs. (a) Radiograph at the initial visit showing a large periapical lesion associated with tooth 45, measuring 14 × 15 mm in diameter. (b) A periapical radiograph of tooth 29 and tooth 30 to observe and record the immediate postoperative situation. (c) Radiograph after 3 months showing a decrease in the size of the periapical radiolucency to 13 × 14 mm in diameter and some material implanted seemed to be absorbed. (d) Radiograph after 7 months showing an apparent decrease in the size of the radiolucent area to 4 × 5 mm in diameter.

Clinical examination revealed obvious gingival swelling over the buccal region of tooth 46 (Figure 1(a)). The tooth was mildly uncomfortable to percussion with a normal response to thermal testing and no any apparent occlusal problems. The probing depths for all sites in the dentition did not exceed 3 mm except for a 10-mm pocket in the buccal furcation of tooth 46 (Figure 1(b)). However, an obvious worn-down abnormal central cusp was found on the occlusal central fossa of tooth 45, which had obvious discomfort on percussion, no response to thermal testing, and no mobility. The radiograph showed a well-defined round radiolucent lesion approximately 14 × 15 mm in diameter surrounded by a radiopaque line, around the root apex of tooth 45 and with a radiopaque margin involving the apical third of the mesial root of tooth 46 (Figure 2(a)). A small area of radiolucency was also present in the furcation of tooth 46 (Figure 2(a)). No obvious radiolucent lesions were seen elsewhere in the dentition. Based on the clinical history and clinical and radiographic examination, an initial diagnosis of a periapical cyst of tooth 45 accompanied by acute abscess draining through the periodontal ligament of tooth 46 was made.

### 2.2. Treatment

Initially, tooth 45 was treated with routine endodontic therapy. Both conservative and surgical treatment options were explained to the patient and he insisted on the surgical one for his limited time. A consent form was signed by the patient and the surgery was scheduled for the next visit.

**Figure 3:**
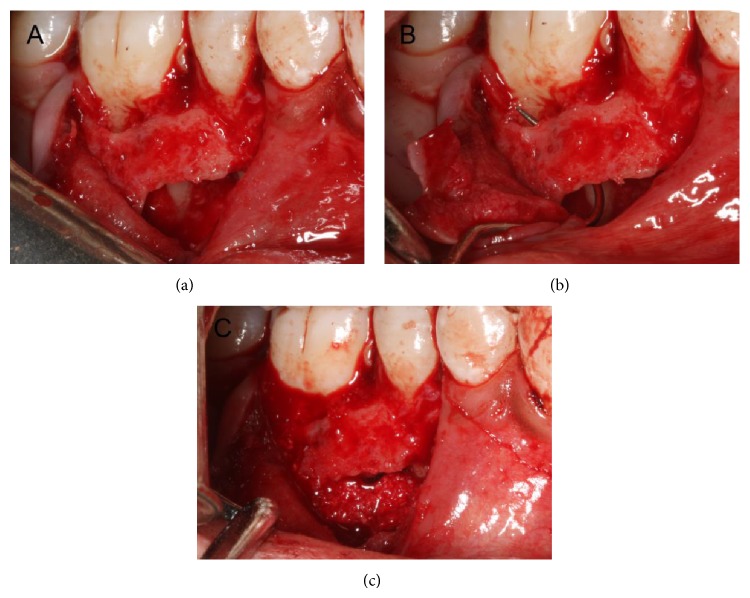
Intraoperative photographs. (a) Osseous destruction region did not involve the root apex of tooth 30. (b) Osseous destruction region around the root apex of tooth 29 penetrated through periodontal ligament of the mesial root and involved the furcation area of tooth 30. (c) Osseous destruction region was filled with Bio-Oss Collagen.

At the second visit, periapical surgery was done. After standard disinfection, the operative region was anesthetized by block injection of Lidocaine HCl and infiltration injection of Primacaine Adrenaline (articaine hydrochloride with epinephrine tartrate injection). A relieving vertical incision was made on the distobuccal axial angle of tooth 44 and then a crevicular incision was made from tooth 45 to 46 along the buccal gingival sulcus. A full-thickness mucoperiosteal flap was reflected. The tissue of cystiform around tooth 45 was enucleated and then a 15 × 12 × 8 mm osseous destruction region was exposed. Further inspection found that the osseous destruction around the root apex of tooth 45 penetrated through periodontal ligament of the mesial root and involved the furcation area of tooth 46 (Figures 3(a) and 3(b)). Then, root resection and root-end filling of tooth 45 were done and granulation tissue was removed thoroughly. After the surgical region was rinsed with normal saline, the bony wall of the cavity was scratched until it was filled with blood, and then Bio-Oss Collagen was placed into the bony destruction of teeth 45 and 46 (Figure 3(c)), as has been previously suggested [16]. Finally, the mucoperiosteal flap was repositioned and the surgical region was closed with 4-0 vicryl sutures. A periapical radiograph of teeth 45 and 46 was taken to observe and record the immediate postoperative situation (Figure 2(b)). After the procedure, the patient was advised to take amoxicillin (1500 mg per day) and ornidazole (1000 mg per day) orally for five days and gargle with 0.12% chlorhexidine gluconate for one week. A week later, the patient returned for postoperative examination and suture removal.

### 2.3. Prognosis

At 3 and 7 months of recall, clinical and radiographic examinations were performed to evaluate the healing status. At 3 months, oral examination revealed good healing of soft tissues and the furcal probing depth of tooth 46 had lessened to 4 mm (Figure 1(c)). A radiograph revealed a decrease in the size of the periapical radiolucency to 13 × 14 mm in diameter and some material implanted seemed to be absorbed (Figure 2(c)). At 7 months, the soft tissues were in healthy condition and the furcal probing depth of tooth 46 was about 2 mm (Figure 1(d)). A radiograph showed an apparent decrease in the size of the radiolucent area to 4 × 5 mm in diameter (Figure 2(d)). Additionally, tooth 45 had normal response to thermal testing at all appointments.

## 3. Discussion

A sinus tract stoma of periapical inflammation is usually opposite to the causative teeth but occasionally may have some distance from the involved teeth, which often produces a diagnostic dilemma. Here, a rare case with an unusual drainage route has been reported in which the gingival sulcus of an adjacent tooth became a stoma of the sinus tract for periapical inflammation.

Such cases as presented above have been rarely reported. To our limited knowledge, only Kelly and Ellinger [11] reported 4 ones. As for these cases, diagnostic evidences indicating that the gingival sulcus of an adjacent tooth was the drainage stoma for periapical inflammation were mainly based on the radiographic and clinical examinations and relevant treatment outcomes. In the present case, the periodontium of the patient was overall healthy except for the buccal surface of vital tooth 46. And the buccal bone destruction of tooth 46 exposed during the surgery was interlinked with the cystic cavity of tooth 45. Additionally, complete recovery of deep periodontal pocket was obtained after root canal treatment and periapical surgery with Bio-Oss Collagen implantation for tooth 45. Based on the above facts, it was reasonable to consider that the deep periodontal pocket of tooth 46 originated from apical periodontitis of tooth 45. Hence, formation process of the sinus tract could be that the periapical inflammation first destroyed tissues around the root apex of tooth 45, then involved the buccal periodontal tissues continuous with the apical third of tooth 46, and finally spread along its periodontal ligament directly to form an ectopic sinus tract stoma on the gingival sulcus. It is the first time to directly observe the specific drainage route.

Given that inflammatory exudates commonly spread to lower site following the law of gravity, gravity is naturally believed to exert some influence on the discharging direction of periapical infection and has been regarded as an explanation for extraoral drainage routes [17–19]. As for this case, tooth 46 is in the distal site of tooth 45 and lower than tooth 45, especially in the supine position. Therefore, the infection spreading from tooth 45 to tooth 46 conforms with the law of gravity. Kelly and Ellinger [11] reported four cases similar to the present one: the first case demonstrated periapical infection of tooth 36 exiting through the periodontal ligament of tooth 37; the second one presented apical periodontitis of tooth 21 resulting in a sinus tract stoma on the gingival sulcus of tooth 22; then the third one performed apical periodontitis of tooth 46 to form a sinus opening on the gingival sulcus of tooth 47; and the final case showed periapical inflammation of tooth 15 draining through the periodontal ligament of tooth 16. Obviously, the above cases also accord with this law that the uninvolved teeth on which the sinus stomas lie are all in the distal site of the causative teeth. Thus, gravity may be a possible explanation for the drainage sinus formation in the present case.

Periapical infection commonly breaks through the nearest cortical plate of the alveolar bone, penetrates the periosteum, and further spreads through the contiguous tissues to form various sinus tracts [20]. As barriers against the inflammation, bone, muscles, periodontal ligament, and so forth certainly play a major role in the drainage direction of periapical infection [8, 18, 19, 21]. In the maxilla, the bony thickness from the root apex to the alveolar cortical plate surface was markedly thinner on the facial side than that on the palatal side [17, 21], while in the mandible, its thinner and weaker parts lie in the labial side of anterior teeth and lingual side of the molar region [8, 17]. These may partially explain the clinical phenomenon that fistulae commonly exit through facial surface for both maxillary teeth and mandibular anterior teeth, but through lingual surface for mandibular posterior teeth. Reports also indicated that if the infection penetrated the cortical plate proximal to muscular attachments, it would usually erupt into the oral vestibule, but if beyond its muscle insertions, the infection would probably travel out from the oral cavity to form various sinus stomas away from the causative teeth [17, 18, 21]. Hence, muscle attachment sites to the bone can sometimes affect the spreading of periapical infections. Meanwhile, preexisting periodontal lesion including breakdown of connective tissues and even bone resorption will generally lower the defense ability of the periodontal tissues against the infection [11]. Suppuration may discharge along the destructed periodontal tissues and exit through the gingival sulcus or periodontal pocket of the tooth with periodontal lesion, rather than gingival or vestibular mucosa opposite to the causative tooth [10, 11, 15].

The causative tooth itself with its various anatomical features should also be responsible for the variety of the drainage pathways [8, 11, 17, 18, 21]. In the maxilla, the roots of the premolars and molars usually lie in close relation to the maxillary sinus, so infections from these teeth frequently involve the maxillary sinus [17, 20, 21]. In mandible, the facial sinus on the chin or submental point is almost inevitably due to infections originating from the mandibular anterior teeth [17, 22]. Consequently, the different anatomical position of the causative teeth may result in distinct sinus tracts. However, for the same tooth, roots with different length also can have different relationship to their contiguous structures [8, 17, 18], forming diverse sinus stomas. For instance, infections caused by the tooth with a longer root have a greater chance to form a bony breakthrough beyond the muscular attachments, usually resulting in an extraoral sinus stoma, while those from the tooth with shorter root have fewer chances and commonly tend to form an intraoral stoma [8, 17, 18]. Moreover, interradicular root proximity, often caused by dilacerations, may make the periapical infection easily penetrate the thinner tissues between the causative tooth and its adjacent tooth and then spread to periodontal tissues of the adjacent tooth, especially for some extreme instances in which the causative tooth partly shares a common cementum (concrescence) or periodontal ligament with its adjacent tooth [11]. In the present case, root proximity may be another probable explanation for this ectopic drainage, considering that the root apex of tooth 45 bends to distal orientation slightly, making the tissues between the root apex of tooth 45 and tooth 46 thinner and the inflammation easier to involve tooth 46.

Modern endodontic concept commonly insisted that conservative treatment should be the first choice for a periapical disease. But in the present case, surgical treatment was first chosen considering the patients' demands and initial diagnosis. Despite the fact that the treatment had certain defects, ideal treatment effect has been received and more importantly, this paper mainly aims to show a rare drainage route of periapical inflammation and elucidate the factors determining the diversity of discharging routes of periapical inflammation.

## 4. Conclusions

The sinus tracts for periapical inflammation are various and are not always found in stomas exactly opposite to the causative teeth, which have resulted in numbers of misdiagnostic cases. Healthy gingival sulcular tissue may become a stoma for the sinus tract of periapical inflammation of an adjacent tooth which manifests as a lesion resulting from periodontal disease. Different drainage pathways of periapical inflammation are mainly related to factors including gravity, barriers against the inflammation, and the causative tooth itself.
